# *N* ^2^-Alkyl-dG lesions elicit R-loop accumulation in the genome

**DOI:** 10.1093/nar/gkae845

**Published:** 2024-10-01

**Authors:** Yinan Wang, Feng Tang, Ting Zhao, Jun Yuan, Andrew H Kellum, Yinsheng Wang

**Affiliations:** Department of Chemistry, University of California, Riverside, CA 92521-0403, USA; Department of Chemistry, University of California, Riverside, CA 92521-0403, USA; Environmental Toxicology Graduate Program, University of California, Riverside, CA 92521-0403, USA; Environmental Toxicology Graduate Program, University of California, Riverside, CA 92521-0403, USA; Department of Chemistry, University of California, Riverside, CA 92521-0403, USA; Department of Chemistry, University of California, Riverside, CA 92521-0403, USA; Environmental Toxicology Graduate Program, University of California, Riverside, CA 92521-0403, USA

## Abstract

Humans are exposed to DNA alkylating agents through endogenous metabolism, environmental exposure and cancer chemotherapy. The resulting alkylated DNA adducts may elicit genome instability by perturbing DNA replication and transcription. R-loops regulate various cellular processes, including transcription, DNA repair, and telomere maintenance. However, unscheduled R-loops are also recognized as potential sources of DNA damage and genome instability. In this study, by employing fluorescence microscopy and R-loop sequencing approaches, we uncovered, for the first time, that minor-groove *N*^2^*-*alkyl-dG lesions elicit elevated R-loop accumulation in chromatin and in plasmid DNA in cells. We also demonstrated that the *N*^2^*-*alkyl-dG-induced R-loops impede transcription elongation and compromise genome integrity. Moreover, genetic depletion of DDX23, a R-loop helicase, renders cells more sensitive toward benzo[*a*]pyrene diolepoxide, a carcinogen that induces mainly the minor-groove *N*^2^-dG adduct. Together, our work unveiled that unrepaired minor-groove *N*^2^-alkyl-dG lesions may perturb genome integrity through augmenting R-loop levels in chromatin. Our findings suggest a potential therapeutic strategy involving the combination of R-loop helicase inhibitors with DNA alkylating drugs.

## Graphical Abstract

**Graphical Abstract:**
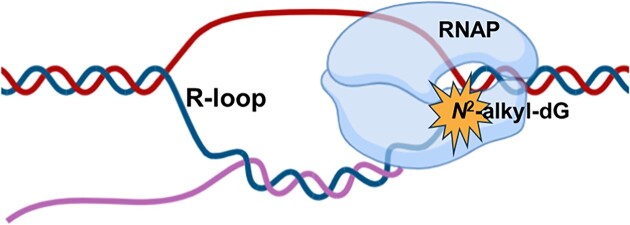

## Introduction

Alkylating agents are generated from cellular metabolism, widely present in environmental pollutants, and commonly used in cancer chemotherapy (1,2). Exposure to those agents induces DNA damage in living cells, and the ensuing DNA adducts may result in genome instability by impeding DNA replication and transcription (1,3). A number of alkylating agents can react with the *N*^2^ of 2′-deoxyguanosine (dG) in DNA to form *N*^2^-alkyl-dG lesions. For example, *N*^2^-(1-carboxyethyl)-2′-deoxyguanosine (*N*^2^-CE-dG) is the major stable adduct formed in DNA upon exposure to methylglyoxal, a byproduct of glycolysis, at physiological concentration and temperature (4,5). Benzo[*a*]pyrene-7,8-dihydrodiol-9,10-epoxide (BPDE), formed from metabolic activation of benzo[*a*]pyrene, reacts predominantly with the *N*^2^ of dG (6). In addition, the *N*^2^ of dG is susceptible to reaction with formaldehyde, which could be induced endogenously, and acetaldehyde, which can be produced from ethanol metabolism or lipid peroxidation, and is also present in external sources, including diesel exhaust, cigarette smoke, etc. (7–9).

R-loops are nucleic acid structures that emerge during transcription when the nascent RNA anneals with the template DNA strand, with a displaced non-template DNA strand (10,11). Initially thought to be rare byproducts of transcription, R-loops are now known to be present throughout the cell cycle in bacteria, yeast, and higher eukaryotes (10,12). There are two types of R-loops, ‘scheduled’ R-loops, which assume functions in normal cellular processes, and ‘unscheduled’ R-loops, formed during periods of cellular dysregulation and associated with replication stress, DNA damage and various human diseases such as neurodegenerative disorders and cancer (10,11). While R-loops are considered a potent source of DNA damage and genome instability, recent studies revealed that DNA double-strand breaks (DSBs) can induce R-loop formation, which facilitates DNA DSB repair (13–15). However, it remains unclear if other DNA lesions also modulate R-loop levels and influence genome integrity in cells.

In light of the previous reports that *N*^2^-alkyl-dG lesions can result in genome instability by hindering transcription (16,17), we hypothesized that these lesions, like DSBs, may also foster elevated accumulation of R-loops in cells. In this study, we examined the contributions of *N*^2^-alkyl-dG lesions to R-loop accumulation. Our results showed, for the first time, that *N*^2^-alkyl-dG lesions trigger increased R-loop levels and lead to genome instability in human cells.

## Materials and methods

### Cell lines

HEK293T cells were purchased from ATCC. All cell lines used in this study were tested to be free of mycoplasma contamination using LookOut Mycoplasma PCR Kit (Sigma, MP0035). Cells were maintained in DMEM (Life Technologies) supplemented with 10% FBS (Invitrogen) and 1% penicillin/streptomycin (v/v) at 37°C in a humidified incubator with 5% CO_2_. For genomic DNA extraction, cells were harvested at 80% confluency. DDX23 and DDX5 knockout cells in HEK293T background were generated using the CRISPR-Cas9 method. [D_9_]-*N*^2^-*n*Bu-dG and *N*^2^-alkyl-dG-bearing oligodeoxyribonucleotides (ODNs) were synthesized following previously published procedures (16,18,19).

### Synthesis and purification of *N*^2^-(6-heptyn)yl-2′-deoxyguanosine (*N*^2^-heptynyl-dG)

*N* ^2^-Heptynyl-dG was synthesized from 2-fluoro-6-*O*-(2-(4-nitrophenyl)ethyl)-2′-deoxyinosine (LGC Biosearch technologies, Inc.) (Supplementary Figure S1). Briefly, 2-fluoro-6-*O*-(2-(4-nitrophenyl)ethyl)-2′-deoxyinosine (4.0 mg, 0.01 mmol) was dissolved in 80 μl dimethyl sulfoxide (DMSO), to which solution was added 6-heptyn-1-amine•HCl (7 mg, 0.05 mmol) and anhydrous *N*,*N*-diisopropylethylamine (DIEA, 3.8 μl, 0.02 mmol). The reaction mixture was stirred at 55°C for 3 days. The resulting mixture was dried *in vacuo*, and reconstituted in 1 M 1,8-diazabicyclo[5.4.0]undec-7-ene (DBU) (100 μl) to deprotect the nitrophenylethyl group. The crude product was dried *in vacuo* and redissolved in 1 mL doubly distilled water.

A Kinetex XB-C18 column (4.6 × 150 mm, 5 μm in particle size and 100 Å in pore size; Phenomenex Inc.) was employed to purify *N*^2^-heptynyl-dG. Doubly distilled water and methanol were selected as mobile phases A and B, respectively. The gradient comprised of 10% B at 0–2 min and 10–80% B at 2–75 min, with the flow rate being 2.5 ml/min. The purified *N*^2^-heptynyl-dG was confirmed by ESI-MS and MS/MS analyses (Supplementary Figure S2).

### Incorporation of *N*^2^-*n*Bu-dG and *N*^2^-heptynyl-dG into genomic DNA

HEK293T cells and the isogenic *DDX23*^−/−^ cells were seeded in 12-well plates with coverslips or 6-well plates at 37°C in a 5% CO_2_ atmosphere. *N*^2^-*n*Bu-dG or *N*^2^-heptynyl-dG were added to the culture medium at a final concentration of 10 μM. After a 3-h incubation, the cells were harvested immediately, or after a 3- or 8-h incubation in fresh media without the modified nucleoside.

### Enzymatic digestion and LC–MS/MS analysis of *N*^2^-*n*Bu-dG and *N*^2^-heptynyl-dG in cellular DNA

Genomic DNA was extracted from cells using Qiagen DNeasy Blood & Tissue Kit, and approximately 6 μg of DNA was recovered from a single well of cells. The extracted genomic DNA was subjected to enzymatic digestion, following previously published procedures (16,20). In brief, 1.0 μg of cellular DNA was digested with 10 units of nuclease P1 and 0.00125 unit of phosphodiesterase II in a buffer with 30 mM sodium acetate (pH 5.6), 1 mM ZnCl_2_, and 2.5 nmol of *erythro*-9-(2-hydroxy-3-nonyl)adenine (EHNA, adenosine deaminase inhibitor). The above mixture was incubated at 37°C for 24 h. To the mixture were then added 1.0 unit of alkaline phosphatase, 0.0025 unit of phosphodiesterase I and one tenth volume of 0.5 M Tris–HCl (pH 8.9). The resulting mixture was incubated at 37°C for another 4 h and subsequently neutralized with 1.0 M formic acid. The enzymes in the digestion mixture were then removed by chloroform extraction. The aqueous phase was dried *in vacuo* and the resulting residues redissolved in water for LC–MS/MS analysis, where an LTQ linear ion-trap mass spectrometer (Thermo Electron, San Jose, CA, USA) and an Agilent Zorbax SB-C18 column (0.5 × 150 mm, 5 μm in particle size) were used. Mobile phases A and B were 2 mM ammonia bicarbonate in doubly distilled water and acetonitrile, respectively. The gradient was 5 min of 5% mobile phase B, followed by 35 min of 5–90% mobile phase B, where the flow rate was 8 μl/min. The temperature for the ion transfer tube was 300°C, and the mass spectrometer was set up to acquire the full-scan MS/MS for the [M + H]^+^ ions of *N*^2^-heptynyl-dG and [D_9_]-*N*^2^-*n*Bu-dG.

### Fluorescence microscopy

Fluorescence microscopy analysis of R-loops was conducted following previously published procedures (21). HEK293T cells, and the isogenic DDX23 and DDX5 knockout cells were cultured on coverslips for 24 h. The cells were subsequently treated with 2 μM BPDE or DMSO control for 3 h. For DDX23 complementation assay, the plasmid for expressing HA-tagged DDX23 protein was kindly provided by Dr Shaochun Yuan (22). The plasmid for expressing the corresponding catalytically dead mutant of HA-tagged DDX23 was constructed by mutating lysine 441 located in the highly conserved Walker A motif of human DDX23 to an asparagine (K441N) (23). The cells in a 6-well plate were transfected the 200 ng of plasmid for wild-type or catalytically dead HA-DDX23. After 24 h, the cells were then fixed with chilled methanol at room temperature for 15 min. The cells were subsequently washed with PBS-TX (PBS containing 0.1% Triton X-100) and blocked with an antibody dilution solution (AbDil-Tx, PBS containing 2% BSA, 0.05% NaN_3_) at 4°C for 6 h. For RNase H treatment, coverslips were incubated in 1 × RNase H buffer (NEB) with 1:50 diluted RNase H (NEB) at 37°C for 3 h. The cells were then incubated with recombinant GFP-tagged catalytically dead RNase H1 (GFP-dRNH1) at 37°C for 1.5 h and washed with PBS-TX. The samples were incubated with 4′,6-diamidino-2-phenylindole (DAPI) (Sigma) for 5 min to stain the DNA and the slides were mounted in Vectashield (Vector Laboratories, Burlingame, CA).

For imaging γ-H2AX, the cells were fixed and blocked following the same procedures as described above. The cells were subsequently incubated with the Phospho-Histone H2A.X (Ser139) antibody (2577S, Cell Signaling) in AbDil-Tx at room temperature for 1.5 h and then with Alexa Fluor 594 goat anti-rabbit IgG (Thermo Fisher). The samples were incubated with DAPI for 5 min to stain the DNA and the slides were mounted in Vectashield. Images were acquired using a Zeiss 880 inverted confocal microscope (Carl Zeiss, Oberkochen, Germany) with a 40×/1.4 oil immersion and quantified using ImageJ. The DAPI channel was used to identify nuclei as the region of interest (ROI). The mean intensity of GFP-dRNH1 within these ROIs was then calculated to yield nuclear mean fluorescence intensities, which reflect nuclear R-loop levels. The *P* values were calculated using two-tailed Student's *t*-test.

### Western blot

Protein samples were separated on an SDS-PAGE gel and transferred to a nitrocellulose membrane (Bio-Rad). After blocking with a blotting-grade blocker (Bio-Rad), the membrane was incubated in a solution containing primary antibody and 5% BSA (w/v) at 4°C overnight, and then incubated with the HRP-conjugated secondary antibody in a 5% blotting-grade blocker (w/v) at room temperature for 1 h. The immunoblots were detected using ECL Western blotting detection reagent (Amersham). Primary antibodies used in this study included α-tubulin (sc-32293, Santa Cruz; 1:10 000), GAPDH (sc-32233, Santa Cruz; 1:10 000), DDX23 (10199-2-AP, Proteintech, 1:2000), DDX5 (67025-1-Ig, Proteintech, 1:2000) and phospho-histone H2A.X (Ser139) (2577S, Cell Signaling Technology; 1:1000).

### Construction of lesion-containing plasmids

The lesion-containing plasmids were constructed according to previously described procedures (24), where 12-mer *N*^2^-alkyl-dG-containing ODNs were employed as the lesion-containing insert. Briefly, the damage-free control vector was digested with Nt.BstNBI to nick the double-stranded parental vector. The 25mer ODN arising from Nt.BstNBI cleavage was subsequently removed by annealing with excess complementary 25mer ODN and by centrifugation using a 100 kDa cutoff centrifugal filter. The 12mer *N*^2^-alkyl-dG-containing ODN (5′-ATGGCGXGCTAT-3′, X = *N*^2^-alkyl-dG) was 5′-phosphorylated and annealed into the gap together with a 13mer 5′-phosphorylated lesion-free ODN (5′-TCGGGAGTCGATG-3′). T4 DNA ligase was then added to seal the gap. The fully ligated, supercoiled plasmid was isolated from the ligation mixture by using agarose gel electrophoresis.

### Cellular transcription, RNA isolation, and RT-PCR

Cellular transcription and RNA isolation, RT-PCR, polyacrylamide gel electrophoresis (PAGE), and LC–MS/MS analysis were carried out as previously described (24). Briefly, the lesion-containing and lesion-free plasmids were individually premixed with the competitor plasmid at a molar ratio of 3:1 (lesion or control/competitor) for transfection. HEK293T, *DDX23*^−/−^ cells, and the latter with ectopic expression of the above-mentioned HA-tagged wild-type DDX23 (1 × 10^5^ cells) were seeded in 24-well plates and cultured overnight at 37°C in a 5% CO_2_ atmosphere, followed by transfection with 50 ng of the mixed plasmids and 450 ng of carrier plasmid (self-ligated pGEM-T, Promega) using TransIT-2020 (Mirus Bio). The cells were harvested at 24 h later, the transcripts of the mixed plasmids were isolated using Total RNA Kit I (Omega), and residual DNA in the mixture was removed with a DNA-free kit (Ambion). The transcripts of interest were reverse-transcribed and PCR-amplified, as described elsewhere (24).

### Restriction digestion, PAGE and LC–MS/MS analysis

For each sample, 150 ng of the above-mentioned RT-PCR product was incubated with 5 U NcoI and 1 U shrimp alkaline phosphatase (rSAP) in 10 μl of 1 × NEB buffer 3.1 at 37°C for 1 h. The enzymes were heat-inactivated by incubation at 70°C for 20 min, and to the mixture were added 5 U T4 polynucleotide kinase and 1.66 pmol [γ-^32^P]ATP to radiolabel the newly liberated 5′-terminus in the template strand. The resultant mixture was heated at 65°C for 20 min and further digested with 2 U SfaNI in 20 μl of 1 × NEB buffer 3.1 at 37°C for 1.5 h. The reaction was terminated with 20 μl of formamide gel-loading buffer (2×), and the DNA mixture was resolved on a 30% native PAGE (acrylamide/bis-acrylamide = 19:1) gel and quantified by phosphorimager analysis. The intensities of the radiolabeled DNA bands were used to calculate the relative bypass efficiency (RBE) with the following equation: RBE (%) = (lesion signal/competitor signal)/(control signal/competitor signal) × 100%, where the competitor signal was employed as the internal standard.

LC–MS and MS/MS were employed to identify unambiguously the transcription products arising from *N*^2^-alkyl-dG-containing templates, as described elsewhere (24). RT-PCR products were treated with 50 U NcoI and 20 U rSAP in 250 μl of NEB buffer 3.1 at 37°C for 2 h, followed by heating at 80°C for 20 min. To the resulting solution was added 50 U SfaNI, and the reaction mixture was incubated at 37°C for 2 h, followed by extraction with phenol/chloroform/isoamyl alcohol (25:24:1, v/v). The aqueous phase was collected, to which were added 0.1 volume of 3.0 M sodium acetate and 2.5 volumes of ethanol to precipitate the DNA. The DNA pellet was reconstituted in water and subjected to LC–MS/MS analysis, where an LTQ linear ion-trap mass spectrometer (Thermo Fisher Scientific) was set up for monitoring the fragmentations of the [M – 3H]^3–^ ions of the 13-mer ODNs.

### Chromatin immunoprecipitation-quantitative PCR (ChIP-qPCR) analysis of R-loops on lesion-bearing plasmids

R-ChIP-qPCR experiments were performed using cells expressing the D210N mutant of V5-tagged RNaseH1 protein, following previously published procedures (25). In brief, cells were seeded in 6-well plates at 37°C in a 5% CO_2_ atmosphere. Lesion-containing or lesion-free plasmids (100 ng) were individually premixed with 900 ng carrier plasmid for transfection. After incubating for 6 h, the cells were washed with cold PBS and cross-linked with 1% formaldehyde at room temperature for 10–15 min. Unreacted formaldehyde was quenched by incubating with 125 mM glycine at room temperature for 15 min. After washing the plates with PBS twice, the cells were scraped off and the nuclei were extracted with a lysis buffer (10 mM Tris–HCl, pH 8.0, 10 mM NaCl, 0.5% NP-40 and 1× protease inhibitor cocktail), and then suspended in a nuclei lysis buffer (50 mM Tris–HCl, pH 8.0, 10 mM EDTA, 1% SDS and 1× protease inhibitor cocktail). Chromatin DNA was sheared to 250–600 bp fragments by sonication. Approximately 5% chromatin fragment was saved as input and the remainder was incubated with magnetic beads conjugated with anti-V5 antibody at 4°C overnight. The beads were washed for three times with washing buffer I (20 mM Tris–HCl, pH 8.0, 150 mM NaCl, 1% Triton X-100, 0.1% SDS, 2 mM EDTA and protease inhibitor cocktail), three times with washing buffer II (the same as buffer I except that the concentration of NaCl was 500 mM), once with washing buffer III (10 mM Tris–HCl, pH 8.0, 250 mM LiCl, 1% NP-40, 1% deoxycholate, 1 mM EDTA and 1 × protease inhibitor cocktail), and then once with TE buffer (10 mM Tris–HCl, pH 8.0 and 1 mM EDTA). The protein-chromatin complex was eluted with an elution buffer (10 mM Tris–HCl, pH 8.0, 1% SDS, and 1 mM EDTA) and cross-links were reversed by incubating at 65°C overnight. After sequential RNase A and Proteinase K treatments, the DNA was purified using Cycle Pure Kit (Omega). Quantitative PCR was performed using standard protocols with Luna® Universal qPCR Master Mix (NEB, M3003X). The data were analyzed using the comparative cycle threshold method (26).

### Strand-specific kethoxal-assisted single-stranded DNA (ssDNA) sequencing (spKAS-seq)

spKAS-seq was conducted following previously published procedures (27) with minor modifications, where *N*^2^-heptynyl-dG was added to the culture medium until its final concentration reached 10 μM. After a 3-h incubation, the cells were treated with 5 mM N_3_-kethoxal (APExBIO Technology LLC, A8793) dissolved in the culture medium at 37°C for 10 min, and then harvested for genomic DNA isolation using the PureLink Genomic DNA Mini Kit (Thermo Fisher Scientific, K182002). The purified genomic DNA was subsequently mixed with 5 μl of 20 mM dibenzocyclooctyne-PEG_4_-biotin (Sigma-Aldrich, 760749) and 10 μl of 10 × phosphate-buffered saline (PBS), and the total volume was adjusted to 100 μl with 25 mM K_3_BO_3_. The resulting mixture was shaken gently at 37°C for 1.5 h, followed by addition of 5 μl of 10 mg/ml RNase A (Thermo Fisher Scientific, 12091039), and the mixture was shaken at 37°C for another 5 min. After the reaction, DNA was isolated using the DNA Clean & Concentrator-5 kit (Zymo Research, D4013) and fragmented by sonication to 150–350 base pairs (bp).

A portion (∼5%) of the above sonicated DNA was saved as input, and the rest was employed for enrichment using 10 μl of Dynabeads MyOne Streptavidin C1 (Thermo Fisher Scientific, 65001). The beads were washed with 1 × binding and washing (B&W) buffer (5 mM Tris–HCl, pH 7.4, 0.5 mM EDTA, 1 M NaCl and 0.05% Tween 20), resuspended in 95 μl of 2 × B&W buffer, and mixed with the sonicated DNA. The suspension was incubated at room temperature for 15 min. The beads were then washed once with 1 × B&W buffer, twice with 100 mM NaOH solution to denature the dsDNA and remove the DNA strands that are not labeled by N_3_-kethoxal, and once again with 1 × B&W buffer. The DNA was eluted from the beads by heating the beads in 10 μl H_2_O at 95°C for 10 min. The ensuing enriched DNA and the corresponding input were employed for library construction using the Accel-NGS Methyl-Seq DNA Library Kit (Swift, 30024).

The sequencing reads of spKAS-seq data were checked with FastQC. Trim Galore was used to remove low-quality base calls and adapter-containing reads from raw spKAS-seq data. Trimmed reads were aligned to the hg19 reference human genome using Bowtie2 with the default setting (28). Sam files were subsequently converted and sorted to binary alignment map (BAM) files using samtools sort (29). Duplicated reads were removed using Picard MarkDuplicates. Browser extended data (BED) files were converted to BedGraph files using bedtools genomecov (30). BedGraph files were then normalized and converted to BigWig files based on the ratio of mapped reads by using ‘KAS-Analyzer normalize’, as described previously (31). Metagene profile plots and heatmaps were generated using deepTools plotProfile and plotHeatmap, respectively (32). Definitions of R-loops by spKAS-seq were performed by following previously reported procedures (27). Two spKAS-seq replicates were employed for R-loop identification. Genomic annotations were performed using Homer (33). The intersection between bed files was performed using BEDTools (30).

### Clonogenic survival assay

Clonogenic survival assay was performed as described previously (34). Briefly, HEK293T and the isogenic *DDX23*^−/−^ cells were plated in triplicate in six-well plates at a concentration of 150 cells per well, Cells were allowed to adhere to the plates for 8 h, and subsequently treated for 9 days with BPDE (0, 0.25, 0.50, 0.75, 1.0, 1.5 μM) (Toronto Research Chemicals Inc., Canada) or *N*^2^-*n*Bu-dG (0, 0.50, 1.0, 2.0, 5.0 and 10 μM). Cell colonies were fixed and stained in an aqueous solution containing 6.0% glutaraldehyde and 0.5% crystal violet. The plates were then rinsed with water and dried at room temperature in air. Colonies were subsequently counted, and survival fraction (SF) was calculated using the following equation: SF = (*N*_clonies, BPDE treated_) / (*N*_clonies, control_).

### *In vitro* transcription and DNA:RNA immunoprecipitation (DRIP) assay

The *in vitro* transcription of lesion-containing plasmids and DRIP assay were performed as described previously (35). *In vitro* transcription reactions were conducted using HeLaScribe® Nuclear Extract *in vitro* Transcription System (Promega) by following the manufacturer's instructions. The reaction mixtures were equally split into two tubes and kept on ice. Five μl of 0.1 mg/ml RNase A (T3018L, NEB) was added to one tube, and to the other tube were added 5 μl of 0.1 mg/ml RNase A and 2 μl RNase H (M0297S, NEB; RNase A + H group). The mixtures were incubated at 37°C for 30 min. Two μl of proteinase K (10 mg/ml) was then added to the mixture, which was incubated at 37°C for 30 min. The nucleic acids were isolated with phenol/chloroform extraction, desalted with ethanol precipitation, and subsequently used for the DRIP assay.

Approximately 5% nucleic acids were saved as input and the remainder was incubated with Pierce™ ChIP-Grade Protein A/G Plus Agarose beads (26159, Thermo Fisher Scientific) conjugated with S9.6 antibody (ENH001, Karafast) at 4°C overnight. The beads were washed three times with DRIP-binding buffer (10 mM sodium phosphate, pH 7.0, 140 mM NaCl and 0.05% Triton X-100). To the mixture was then added 300 μl of the DRIP elution buffer supplemented with 10 μl of proteinase K (10 mg/ml), and the mixture was incubated at 55°C for 45 min with gentle rotation. The supernatant was collected for further purification by phenol/chloroform extraction and ethanol precipitation. The purified DNA was used for qPCR analysis with Luna® Universal qPCR Master Mix. The data were analyzed using the aforementioned comparative cycle threshold method (36). The level of R-loops is represented as the percentage of plasmid DNA recovered after DRIP.

### Real-time quantitative PCR analysis of gene expression levels

Cells were seeded at a 40% confluence and treated with 10 μM *N*^2^-heptynyl-dG for 3 h. Total RNA was extracted from cells using E.Z.N.A.® Total RNA Kit I and used for cDNA synthesis. Approximately 1 μg RNA was reverse transcribed by using SuperScript IV reverse transcriptase (Thermo Fisher) with oligo poly(dT) primer. After a 20 min incubation at 55°C, the reverse transcriptase was deactivated by heating at 80°C for 10 min. Real-time quantitative PCR (RT-qPCR) experiments were performed using Luna® Universal qPCR Master Mix on a Bio-Rad iCycler system, and the running conditions were 95°C for 3 min and 45 cycles of 95°C for 15 s, 55°C for 30 s and 72°C for 45 s. The comparative cycle threshold method (36) was used for the relative quantification of mRNA expression. The levels of gene expression were normalized to *GAPDH*. The sequences of PCR primers are listed in Supplementary Table S1.

## Results

**Figure 1.**
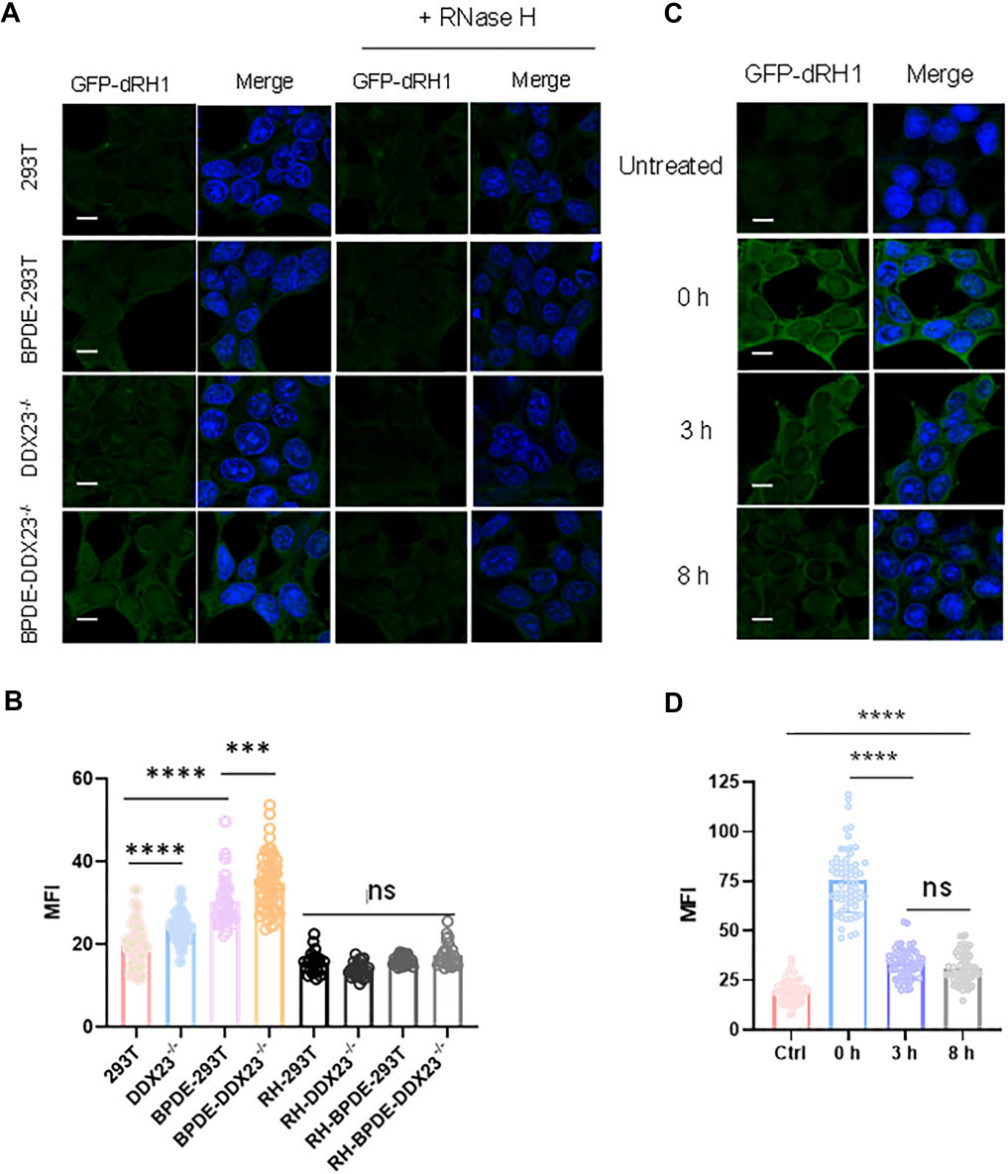
*N* ^2^-alkyl-dG lesions induce elevated R-loop accumulation. (**A**) Fluorescence microscopy analysis of R-loops in HEK293T and the isogenic *DDX23*^−/−^ cells treated with or without BPDE. After fixation, coverslips were treated with or without RNase H, and R-loops were imaged using GFP-dRNH1 (labeled as GFP-dRH1 in the figure). (**B**) Quantification of nuclear R-loop levels, as represented by nuclear mean fluorescence intensities (MFI), for the conditions shown in (A). (**C**) Fluorescence microscopy analysis of R-loops in cells with incorporation of *N*^2^-*n*Bu-dG. After a 3-h exposure to 10 μM of *N*^2^-*n*Bu-dG, the cells were harvested immediately, or cultured in fresh medium without the modified nucleoside for another 3 or 8 h. (**D**) Quantification of nuclear MFI for the conditions shown in c. The data represent the mean ± SD of results obtained from three biological replicates. ns, *P* > 0.05; ***P < 0.001; ****P < 0.0001 (two-tailed Student's *t*-test). Scale bar: 10 μm.

Minor-groove *N*^2^-alkyl-dG lesions are known to impede transcription (16,17). We set out to examine if induction of these lesions could result in elevated R-loop accumulation in cells. To this end, we first assessed if exposure of cells with BPDE, which induces mainly *N*^2^-BPDE-dG, can trigger increased R-loop accumulation by using fluorescence microscopy analysis with GFP-dRNH1 (21). We observed a significant increase in R-loop level after exposure of cells to 2 μM BPDE for 3 h (Figure 1A, B). In addition, genetic ablation of DDX23, which is a R-loop helicase (37), led to a pronounced increase in R-loop levels in BPDE-treated HEK293T cells. Notably, the R-loop signal in DDX23-deficient cells was attenuated upon treatment with recombinant RNase H, suggesting that GFP-dRNH1 signal arises from R-loops in cells. Likewise, we observed augmented R-loop levels in HEK293T cells with another R-loop helicase, i.e. DDX5 (38), being ablated by CRISPR-Cas9 (Supplementary Figure S3).

Next, we sought to determine if other *N*^2^-alkyl-dG lesions could also elicit R-loop formation in chromatin. Spratt and colleagues (39) showed that incubating cultured human cells with *N*^2^-(4-ethynylbenzyl)-dG could allow for facile incorporation of the modified nucleoside into genomic DNA. Hence, we cultured HEK293T cells in a medium containing 2.0 and 10 μM *N*^2^-*n*Bu-dG for 3 h, which facilitated the incorporation of approximately 5 and 15 *N*^2^-*n*Bu-dG per 10^6^ nucleosides into genomic DNA, respectively (16), and assessed the levels of R-loops in cells. We found a significant increase in R-loop levels at 3 h following the treatment, and the extent of increase is in line with the level of *N*^2^-*n*Bu-dG incorporation into genomic DNA (Supplementary Figure S4a–c). In this vein, it is worth noting that treatment with 2 or 10 μM *N*^2^-*n*Bu-dG did not appreciably affect cell survival, as determined by clonogenic survival assay (Supplementary Figure S4d).

A previous study revealed that nucleotide excision repair (NER) contributes to the removal of *N*^2^-*n*BudG from genomic DNA, where a large portion of *N*^2^-*n*Bu-dG in genomic DNA could be repaired within 3 h (16). Hence, we monitored R-loop levels at different time points following removal of *N*^2^-*n*Bu-dG from the culture medium. We found that the R-loop level was significantly attenuated after a 3-h repair compared to 0-h (Figure 1C, D). An 8-h repair led to a slight decrease in R-loop level compared to 3-h repair, which is in line with the previous finding that more than half of *N*^2^-*n*Bu-dG is repaired within 3 h (16). Notably, even after an 8-h repair, R-loops did not restore to the level observed for untreated cells, which is again in agreement with the presence of a small amount of *N*^2^-*n*Bu-dG at this time point (16). Together, these results support that *N*^2^-*n*Bu-dG in genomic DNA stimulates R-loop accrual.

**Figure 2.**
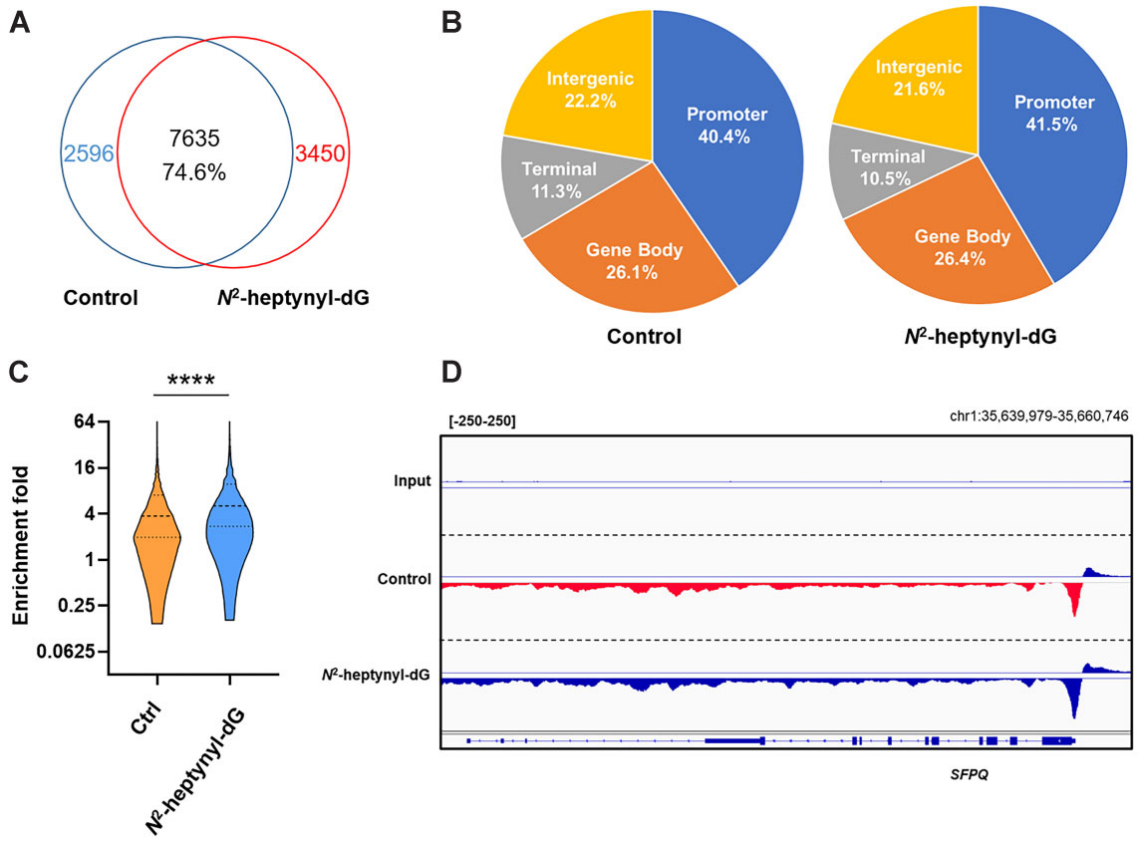
spKAS-seq revealed increased accumulation of R-loops in *N*^2^-heptynyl-dG-treated HEK293T cells. (**A**) A Venn diagram depicting the overlap of R-loop peaks between untreated and *N*^2^-heptynyl-dG-treated HEK293T cells. (**B**) The genomic distribution of R-loop peaks mapped by spKAS-seq. Genomic features are color-coded according to the labels at the bottom. (**C**) Fold enrichment of overlapped peaks between the *N*^2^-heptynyl-dG-treated and untreated cells. ***P < 0.001 (two-tailed Student's *t*-test). (**D**) Representative integrative genomics viewer (IGV) plots displaying the spKAS-seq profile in *N*^2^-heptynyl-dG-treated and control HEK293T cells.

We next conducted spKAS-seq (27) to explore the genome-wide distributions of R-loops in HEK293T cells with or without incorporation of *N*^2^-heptynyl-dG into genomic DNA (Supplementary Figure S5 and S6). Our results showed that, in HEK293T cells, 40.4% and 26.1% of R-loop peaks are located in promoter regions and gene bodies, respectively (Figure 2b), which is in keeping with the previously reported results (27). Treatment with *N*^2^-heptynyl-dG did not appreciably alter the distributions of R-loops, where 41.5% and 26.4% of R-loop peaks were detected in the promoter region and gene bodies, respectively. Surprisingly, R-loop peak numbers only increased slightly from 10231 to 11085 after *N*^2^-heptynyl-dG treatment (Figure 2A). However, when we compared the intensity of those R-loop peaks commonly detected in untreated and *N*^2^-heptynyl-dG-treated cells, R-loop signal exhibits significantly higher intensities in the treated cells (Figure 2C). In this context, the lack of a marked increase in R-loop level is in keeping with the relatively low frequency of incorporation of *N*^2^-heptynyl-dG into genomic DNA, i.e. at 2 lesions per 10^6^ nucleosides (Supplementary Figure S5). Integrative Genomics Viewer (IGV) plots also showed higher R-loop signal intensities in *N*^2^-heptynyl-dG-treated cells than the untreated control (Figure 2D). These results suggest that *N*^2^-heptynyl-dG induces the accumulation of R-loops in those regions that are susceptible to R-loop formation; the lesion, however, does not alter substantially R-loop distribution at the genome-wide scale. Our sequencing results are in line with the previous findings that DSB-induced R-loop formation is not a ubiquitous feature, but rather is restricted to DSBs formed in transcriptionally active regions (40,41).

We and others showed recently that, after metabolic activation, *N*^2^-heptynyl-dG and *N*^2^-(4-ethynylbenzyl)-dG are preferentially incorporated, by DNA polymerase κ, to those genomic regions replicated early in the S phase (42,43). We conducted a correlation analysis of the spKAS-seq data with our recently published *N*^2^-heptynyl-dG sequencing data (42). Such analysis revealed a correlation between loci with *N*^2^-heptynyl-dG-elicited R-loops and sites of *N*^2^-heptynyl-dG incorporation, which are both enriched in regions replicated early in the S phase (Supplementary Figure S7).

**Figure 3.**
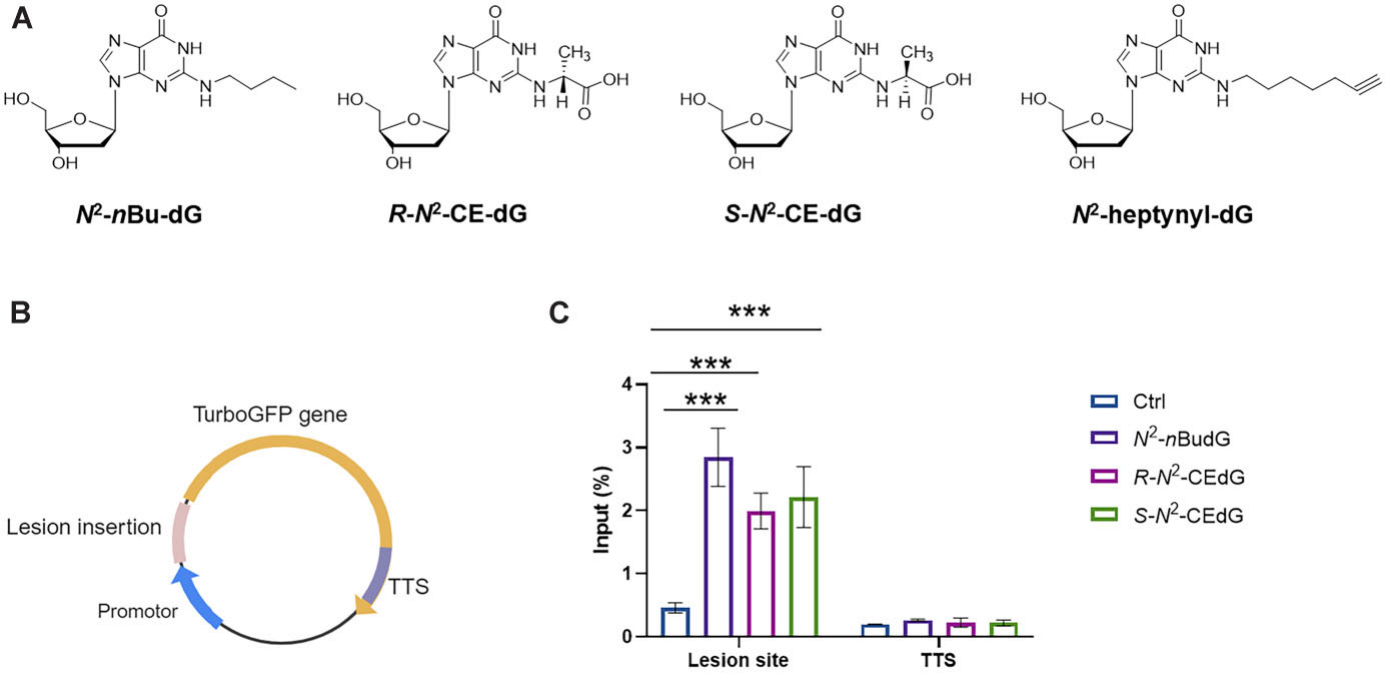
R-loops are accumulated at *N*^2^-alkyl-dG incorporation site. (**A**) Chemical structures of *N*^2^-*n*Bu-dG, *R*-*N*^2^-CE-dG, *S*-*N*^2^-CE-dG, and *N*^2^-heptynyl-dG. (**B**) A schematic diagram showing the lesion-bearing vector, ‘TTS’ indicates the transcription termination site. (**C**) R-ChIP-qPCR analyses in the lesion insertion sites and TTSs. The data represent the mean ± S.D. of results obtained from two biological replicates, where each biological replicate was analyzed twice. ns, *P* > 0.05; ***P < 0.001 (two-tailed Student's *t*-test).

To explore further the induction of R-loops by *N*^2^-alkyl-dG lesions, we constructed plasmids carrying an *N*^2^-alkyl-dG lesion downstream of transcription start site by following previously reported procedures (24). In this vein, we generated plasmids containing an *N*^2^-*n*Bu-dG or the *R* or *S* diastereomer of *N*^2^-CE-dG at a fixed site (Figure 3A and Supplementary Figure S8). We then conducted R-ChIP-qPCR analysis (25) for cells transfected with the *N*^2^-alkyl-dG-bearing plasmids and lesion-free plasmid. It turned out that the R-loop recovery rate was significantly higher at the damage site than the control transcription termination site (Figure 3B). In this vein, all the *N*^2^-alkyl-dG-containing plasmids exhibited a 2-fold higher R-loop recovery rate at the DNA lesion site relative to the damage-free control plasmid, revealing that *N*^2^-alkyl-dG lesions, when situated in transcriptionally active template DNA, triggers augmented R-loop accumulation.

To explore if this sequence is capable of inducing R-loop formation, we conducted the *in-vitro* transcription and DRIP experiments with *N*^2^-alkyl-dG-containing plasmids and a lesion-free control plasmid (35). Our results revealed that RNase A-treated samples exhibit higher recovery rates in the DRIP assay compared to RNase A + RNase H-treated samples (Supplementary Figure S9), indicating the formation of R-loops in the promoter regions of these plasmids. Additionally, the lesion-carrying plasmids exhibited higher recovery rates relative to the lesion-free plasmid (Supplementary Figure S9), supporting the notion that the *N*^2^-alkyl-dG lesions induce increased R-loop accrual. Together, the above results demonstrated that *N*^2^-alkyl-dG lesions induce R-loop accumulation in plasmid DNA.

**Figure 4.**
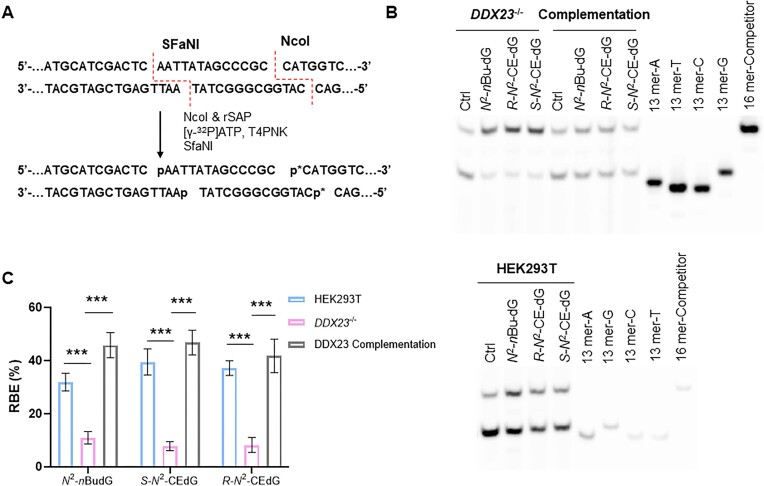
CTAB assay for determining the transcription bypass efficiencies of *N*^2^-alkyl-dG lesions in HEK293T cells, the isogenic *DDX23*^−/−^ cells, and the latter complemented with DDX23. (**A**) A schematic diagram depicting the selective labeling of the template strand via sequential digestion of the RT-PCR products. ‘p*’ denotes a ^32^P-labeled phosphate group. (**B**) Lesion-containing plasmids and the corresponding lesion-free plasmid were individually premixed with the competitor plasmid at a fixed molar ratio of 3:1 (lesion or control/competitor) for transfection into HEK293T cells, *DDX23*^−/−^ cells, and the latter complemented with wild-type DDX23. The transcripts were isolated from cells at 24 h after transfection. The synthetic ODNs are designated as ‘16mer’, which has the same sequence as the restriction fragment from the competitor genome; ‘13 mer-A’, ‘13 mer-T’, ‘13 mer-C’ and ‘13 mer-G’ represent the restriction fragments with A, T, C, and G formed at the lesion site in the PCR product. (**C**) Relative transcription bypass efficiencies (RBEs) of *N*^2^-alkyl-dG lesions. The data represent the mean ± S.D. of results from three independent experiments. ***P < 0.001 (two-tailed Student's *t*-test).

We next asked whether the *N*^2^-alky-dG-induced R-loops near the transcription start site impede transcription. To this end, we conducted the competitive transcription and adduct bypass (CTAB) assay (17) to assess quantitatively how the above-mentioned *N*^2^-alkyl-dG lesions perturb transcription efficiencies in human cells and how the process is modulated by a R-loop helicase. The CTAB method is based on RT-PCR amplification of RNA products emanating from concurrent transcription of a lesion-bearing or lesion-free control plasmids together with a lesion-free competitor vector in cells at a defined molar ratio (Supplementary Figure S8). This is followed by the identification and quantification of restriction fragments released from the RT-PCR products by polyacrylamide gel electrophoresis (PAGE) and LC–MS/MS analyses, as detailed in Materials and Methods (Figure 4a, b). In this vein, the competitor plasmid serves as an internal reference to gauge the degree to which a structurally defined DNA lesion impedes transcription in cells (44). Our results showed that the presence of *N*^2^-alkyl-dG lesions on the transcribed strand led to pronouncedly diminished transcription bypass efficiencies in HEK293T cells (Figure 4C), which is in line with previous observations made for these lesions (16,17,45,46). Importantly, genetic ablation of DDX23 in HEK293T cells led to stronger diminutions in transcription efficiencies of the *N*^2^-alkyl-dG-bearing templates. Moreover, complementation of *DDX23*^−/−^ cells with wild-type DDX23 partially rescues the transcription suppression elicited by *N*^2^-alkyl-dG lesions. In this vein, PAGE, LC–MS and MS/MS analyses revealed that ablation of DDX23 does not lead to ribonucleotide misinsertions opposite the *N*^2^-alkyl-dG lesions (Figure 4B, Supplementary Figures S10, and S11). We next explored the effect of the lesion-induced R-loops on gene expression in human cells by performing RT-qPCR experiments for several selected genes (i.e. *KBTBD6*, *ENTPD5* and *CHERP*), where spKAS-seq revealed elevated R-loops in the promoter regions after *N*^2^-heptynyl-dG treatment. Our results showed diminished expression of these genes, whereas no apparent change in expression was observed for a control gene (*CLSPN*) without alteration in R-loop level after *N*^2^-heptynyl-dG exposure (Supplementary Figure S12). These findings suggest that *N*^2^-alkyl-dG lesions trigger R-loop accumulation, which impedes transcription.

**Figure 5.**
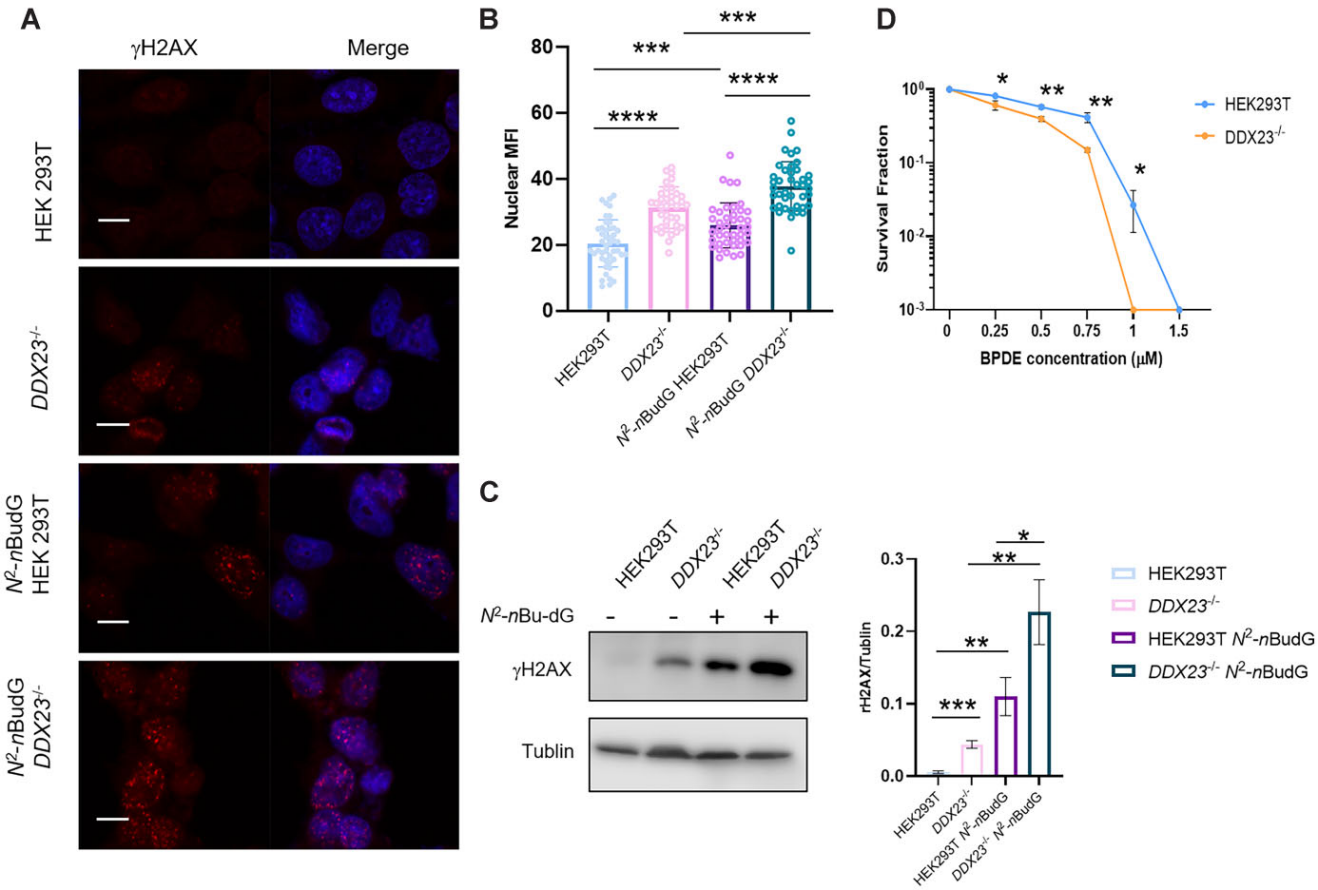
Incorporation of *N*^2^-alkyl-dG into genomic DNA elicits genome instability. (**A**) Representative images of γ-H2AX foci in HEK293T and *DDX23*^−/−^ cells treated with or without *N*^2^-*n*Bu-dG. (**B**) Quantification of nuclear γ-H2AX mean fluorescence intensities (MFI) for the conditions shown in (A). The data represent the mean ± S.D. of results obtained from three biological replicates. ***P < 0.001; ****P < 0.0001 (two-tailed Student's *t*-test). Scale bar: 10 μm. (**C**) γ-H2AX protein levels in whole-cell lysates of parental HEK293T and the isogenic *DDX23*^−/−^ cells (*n* = 3). (**D**) Clonogenic cell survival assay results for HEK293T and the isogenic *DDX23*^−/−^ cells upon BPDE treatment. Cells were plated, and the number of colonies was counted 9 days later. The surviving fractions (SF) were calculated after normalizing to the plating efficiency of the cells without BPDE treatment. The data represent the mean ± S.D. of results from three independent experiments. *, 0.01 < *P* < 0.05; **, 0.001 < *P* < 0.01, ***, *P* < 0.001 (two-tailed Student's *t*-test).

Next, we sought to test whether the *N*^2^-alkyl-dG-induced R-loops contribute to genome instability. In replicating cells, R-loops are known to impede the progression of replication forks and induce DNA DSBs (47). γH2AX is a sensitive molecular marker of DNA damage and accumulates at DNA DSB sites (48). We observed significantly higher γH2AX immunofluorescence signal intensity in *DDX23*^−/−^ cells compared to parental HEK293T cells, which could be rescued by ectopic expression of wild-type DDX23, but not its helicase-defective mutant (Figure 5A, B, Supplementary Figure S13). These results suggest that R-loop accumulation may induce DSBs. In addition, we detected elevated γH2AX signal in *N*^2^-*n*Bu-dG-treated cells compared to the untreated control, where the γH2AX signal was further augmented in *DDX23*^−/−^ cells. These observations are corroborated by results obtained from western blot analysis (Figure 5C). Together, our findings support that *N*^2^-alkyl-dG lesions induce R-loop accumulation, which further exacerbates genome instability.

Finally, we conducted a clonogenic survival assay to examine how R-loop accumulation modulates cellular sensitivity toward BPDE. As expected, BPDE treatment alone exerts a strong influence on impeding colony formation (Figure 5D). Exposure to 0.75 μM BPDE reduced the number of colonies by 80–90%, while a concentration of 1.5 μM completely inhibited colony growth. Genetic ablation of DDX23 strongly sensitized cells to BPDE exposure, where fewer colonies were formed from cells treated with different concentrations of BPDE compared to parental HEK293T cells (Figure 5D).

## Discussion

R-loops are dynamic nucleic acid structures formed during transcription when the nascent RNA forms a heteroduplex with the template DNA strand, leaving a displaced single-stranded DNA (10,11). R-loops are recognized as sources of DNA damage, and they lead to genome instability, though the underlying mechanisms are still under investigation (10,11). Accordingly, unscheduled R-loops confer various pathological consequences, including mutagenesis, replication stress, increased genomic instability, and promotion of cancer development (10,11). On the other hand, R-loops also assume physiological roles by regulating cellular processes, e.g. transcription, chromatin structure, DNA repair, and telomere maintenance in physiological contexts (10,11). Although R-loops are known to result in DNA damage, recent studies revealed that DNA DSBs can induce increased R-loop levels (13–15). It remains unclear whether other DNA lesions can also elicit elevated R-loop accumulation.

In this study, we demonstrated, for the first time, that minor-groove *N*^2^-alkyl-dG lesions trigger R-loop accumulation in human cells. Recently, NER pathway was shown to be responsible for removing adducts formed at the *N*^2^ position of dG (17,49). By using fluorescence microscopy analysis, we showed that different *N*^2^-alkyl-dG lesions, i.e. *N*^2^-BPDE-dG and *N*^2^-*n*Bu-dG, can elicit R-loop accrual in HEK293T cells, where the levels of R-loops induced are positively correlated with the frequencies of *N*^2^-*n*Bu-dG in genomic DNA (Figure 1 and Supplementary Figure S4). Upon repair of *N*^2^-*n*Bu-dG, the R-loop signal decreased, substantiating that these R-loops are induced by *N*^2^-alkyl-dG.

To explore this effect at a genome-wide scale, we performed R-loop sequencing of HEK293T cells with or without *N*^2^-heptynyl-dG treatment. Our results revealed augmented R-loop signal intensities in those genomic regions replicated early in the S phase and already enriched with R-loops, though no significant induction of new R-loops in other regions was observed (Figure 2). These results suggest that *N*^2^-alkyl-dG-induced R-loop formation is not a ubiquitous feature, but rather limited to *N*^2^-alkyl-dG lesions formed in transcriptionally active genomic regions. Our results are in line with the previous findings that the accumulation of DNA/RNA hybrids in *cis* to DSBs induced by enzymatic cleavage at loci occupied by RNAPII before damage, but not around DSBs induced in intergenic loci despite equivalent levels of DSB induction (40,41). These results support a ‘transcription regulation’ model, where lesion-induced R-loops may form as a consequence of transcriptional repression occurring in *cis* to *N*^2^-alkyl-dG lesion, rather than as a consequence of *de novo* transcription at lesion sites.

We further validated the *N*^2^-alkyl-dG-induced R-loop accumulation by incorporating several different *N*^2^-alkyl-dG lesions near the transcription start site of a plasmid. R-ChIP-qPCR results showed that R-loop signal is increased significantly in those *N*^2^-alkyl-dG lesion-containing plasmids compared to the control (Figure 3), supporting that R-loop accumulation is directly induced by *N*^2^-alkyl-dG lesions in human cells. We also employed these plasmids to examine the effects of *N*^2^-alkyl-dG-induced R-loops on transcription efficiency by virtue of the CTAB assay (Figure 4). Our results showed that *N*^2^-alkyl-dG lesions impede transcription, which is consistent with previous studies (16,17,45,46). Genetic depletion of DDX23, a helicase that unwinds R-loop structure (37), further attenuated transcription efficiency, indicating that the lesion-induced R-loops contribute to diminished transcription across the lesion site. These results are supportive of a model, where *N*^2^-alkyl-dG lesions inhibit transcription elongation and induce R-loop accrual, which further attenuates transcription and triggers genome instability. This notion finds its support by a recent observation that R-loops contribute to polycomb repressive complex 1 recruitment and transcriptional repression of polycomb-repressed gene at the genome-wide scale (50).

We also explored the contribution of *N*^2^-alkyl-dG lesion-induced R-loops to genome instability. Our immunofluorescence and Western blots results revealed elevated formation of DSBs after R-loop accumulation elicited by *N*^2^-alkyl-dG (Figure 5). In addition, our clonogenic survival assay revealed that genetic ablation of DDX23, a R-loops helicase, exacerbated the cytotoxicity of the *N*^2^-BPDE-dG (Figure 5). These results are consistent with a previous study indicating that unscheduled R-loops are actively processed into DSBs by NER endonucleases XPF and XPG (47).

In summary, we demonstrated that alkylated DNA lesions formed at the minor-groove *N*^2^ position of dG elicit R-loop accumulation in chromatin. In addition, the *N*^2^-alkyl-dG-induced R-loops impede transcription and contribute to genome instability. Our results suggest a potential therapeutic strategy through the combination of R-loop helicase inhibitors with DNA alkylating anticancer drugs. It would be interesting to explore, in the future, whether *N*^2^-alkyl-dG lesion-induced R-loops facilitate the repair of these lesions, whether the lesion-elicited R-loop accumulation occurs through elevated formation and/or diminished resolution, and if other lesions can also promote the formation of R-loops.

## Supplementary Material

gkae845_Supplemental_File

## Data Availability

The sequencing data generated in this study have been deposited into the NCBI Gene Expression Omnibus (GEO) under accession number GSE 248210.
