# Supplementary material for: N 2-Alkyl-dG lesions elicit R-loop accumulation in the genome
Source: Nucleic Acids Res. 2024 Oct 1;52(20):12487–97. doi: 10.1093/nar/gkae845 (PMC11551765; doi:10.1093/nar/gkae845)
Supplement: gkae845_Supplemental_File [file gkae845_supplemental_file.pdf]

Supplementary Materials for

“N^2-alkyl-dG lesions Elicit R-loop Accumulation in the Genome”

Yinan Wang[1,†], Feng Tang[1,†], Ting Zhao[2], Jun Yuan[2], Andrew H. Kellum, Jr.[1], and Yinsheng Wang[1,2,*]

[1]Department of Chemistry and [2]Environmental Toxicology Graduate Program, University of California, Riverside, CA92521-0403, USA. [†]These authors contributed equally.

*Corresponding authors: Yinsheng Wang.

E-mail: Yinsheng.Wang@ucr.edu

Table of Contents:

Contents	Pages
**Table S1.** Oligonucleotides used in this study.	**S2**
**Figure S1.** A Scheme showing the chemical synthesis of N^2-heptynyl-dG.	**S3**
**Figure S2.** ESI-MS and MS/MS characterizations of N^2-heptynyl-dG.	**S4**
**Figure S3.** N^2-alkyl-dG lesions induce elevated R-loop accumulation in DDX5-deficient cells.	**S5**
**Figure S4.** A higher level of N^2-nBu-dG incorporation confers a higher level of R-loop accumulation.	**S6**
**Figure S5.** N^2-heptynyl-dG incorporation assay.	**S7**
**Figure S6.** Representative selected-ion chromatograms (SICs) and MS/MS [D$_9$]-N^2-*n*Bu-dG and N^2-heptynyl-dG.	**S8**
**Figure S7.** N^2-heptynyl-dG and elevated R-loops are co-enriched in genomic regions replicated in the early S phase of the cell cycle.	**S9**
**Figure S8.** A schematic diagram showing the workflow of the CTAB assay.	**S10**
**Figure S9.** DRIP-qPCR results.	**S11**
**Figure S10.** Higher resolution ‘ultra zoom-scan’ ESI-MS for monitoring the $[M-3H]^{3-}$ ions of the restriction fragments of interest from transcription of non-lesion control and N^2-alkyl-dG lesions.	**S12**
**Figure S11.** LC-MS/MS for monitoring the restriction digestion products of interest.	**S13**
**Figure S12.** Representative IGV plots and RT-qPCR results for selected genes in HEK293T cells with and without N^2-heptynyl-dG treatment.	**S14**
**Figure S13.** Deficiency in DDX23 elicits genome instability.	**S15**

Table S1. Oligonucleotides used in this study.

Primer names	Sequences
Lesion insertion site qPCR forward	5′-CAGAGCTGGTTTAGTGAACCGT-3′
Lesion insertion site qPCR reverse	5′-GGTACCGTCGACTGCAGAAT-3′
Plasmid TTS qPCR forward	5′-AGGATCACAGCAACACCGAG-3′
Plasmid TTS qPCR reverse	5′-TTCTTCACCGGCATCTGCAT-3′
CTAB assay reverse transcription PCR	5′-TCGGTGTTGCTGTGAT-3′
CTAB assay amplification PCR forward	5′-CTAGCGGATGCATCGACTC-3′
CTAB assay amplification PCR reverse	5′-TGCTGCGGATGATCTTGTCG-3′
KBTBD6-F	5′-CAGTTAGGGCAGAATGGAGG-3′
KBTBD6-R	5′-ATA TTC ATA CAC AGT CAC CCG G-3′
ENTPD5-F	5′-AGT GCC TGT TTA CCG AGA TG-3′
ENTPD5-R	5′-TGG TGA AGT TTT CCT CGT ACC-3′
CHERP-F	5′-AAA CGG CTA CTT CGA TGA CTC-3′
CHERP-R	5′-GTC TTG AGG GTC TGG ATC TG-3′
CLSPN-F	5′-TGG TAA TGA TCT GGC ACT GG-3′
CLSPN-R	5′-TCA TCT TCG CTT CCC ACA TC-3′
GAPDH-F	5′-ACA TCG CTC AGA CAC CAT G-3′
GAPDH-R	5′-TGT AGT TGA GGT CAA TGA AGG G-3′
DDX23_K441N-F	5′-GCTGAGACTGGCAGTGGCAA*C*ACAGCAGC-3′
DDX23_K441N-R	5′-CTGCCAGTCTCAGCCACACCAATGATGTCACGA-3′

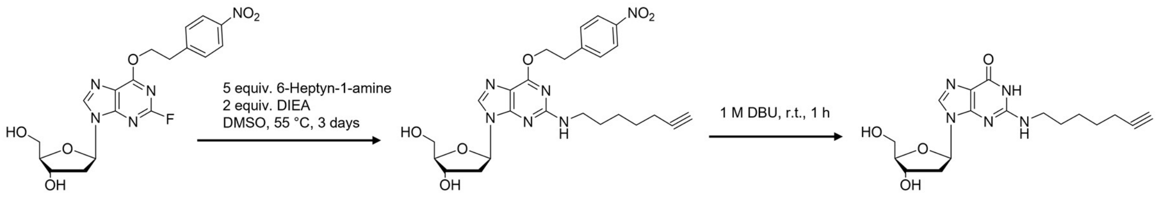

Figure S1. A Scheme showing the chemical synthesis of N^2-heptynyl-dG.

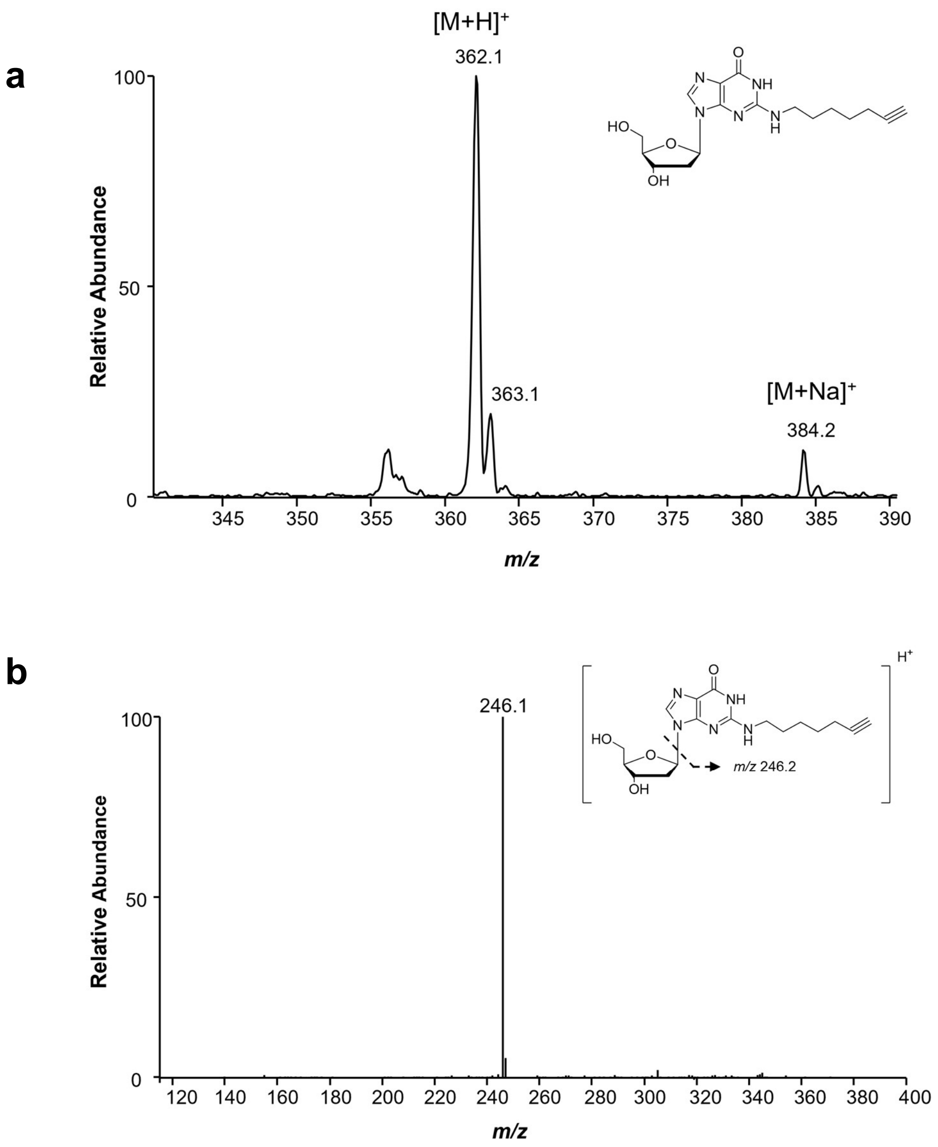

Figure S2. Positive-ion ESI-MS (a) and MS/MS (b) of N^2-heptynyl-dG.

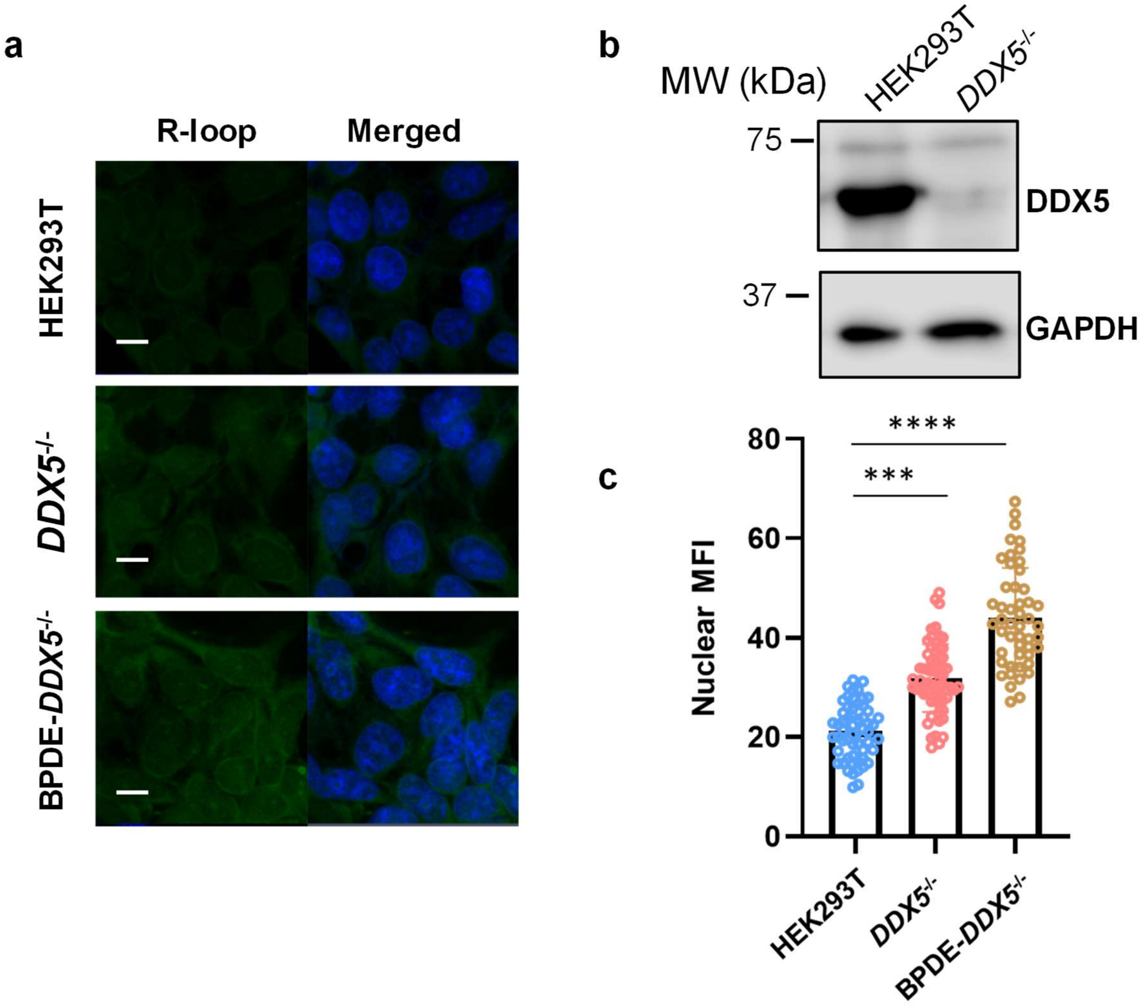

Figure S3. Genetic ablation of DDX5 led to elevated R-loop accumulation, which is exacerbated by BPDE treatment. (a) Fluorescence microscopy analysis of R-loops in HEK293T cells, and the isogenic *DDX5*$^{-/-}$ cells with or without a 3-h exposure to 2.0 μM BPDE. (b) Western blot for confirming the successful knock-out of DDX5 in HEK293T cells. (c) Quantification of nuclear R-loop levels, as represented by mean fluorescence intensities (MFI), for the conditions shown in (a). The data represent combined results from three biological replicates. ***, $p < 0.001$; ****, $p < 0.0001$ (two-tailed Student's *t*-test). Scale bar: 10 μm.

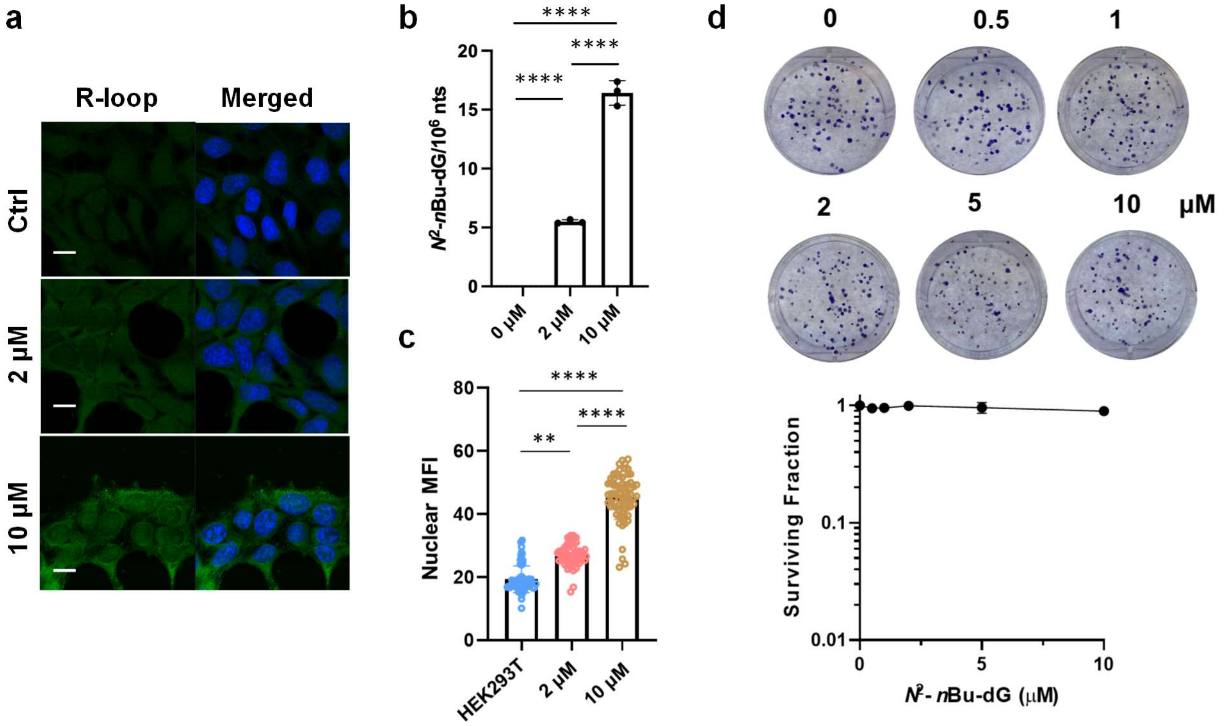

Figure S4. A higher level of N^2-*n*Bu-dG incorporation into genomic DNA confers a higher level of R-loop accumulation. (a) Fluorescence microscopy analysis of R-loops in HEK293T cells treated with different concentrations of N^2-nBu-dG for 3 h. (b) Frequencies of N^2-*n*Bu-dG in cellular DNA isolated from HEK293T cells treated with different concentrations of N^2-*n*Bu-dG for 3 h. (c) Quantification of nuclear R-loop levels, as reflected by nuclear mean fluorescence intensities (MFI), for the conditions shown in (a). The data represent combined results from three biological replicates. **, $p < 0.01$; ***, $p < 0.001$; ****, $p < 0.0001$ (two-tailed Student's *t*-test). Scale bar: 10 μm. (d) Clonogenic survival assay results for HEK293T treated with the indicated concentrations of N^2-*n*Bu-dG. The surviving fractions (SF) were calculated after normalizing to the plating efficiency of the cells without N^2-*n*Bu-dG treatment. The data represent the mean ± S.D. of results from three independent experiments.

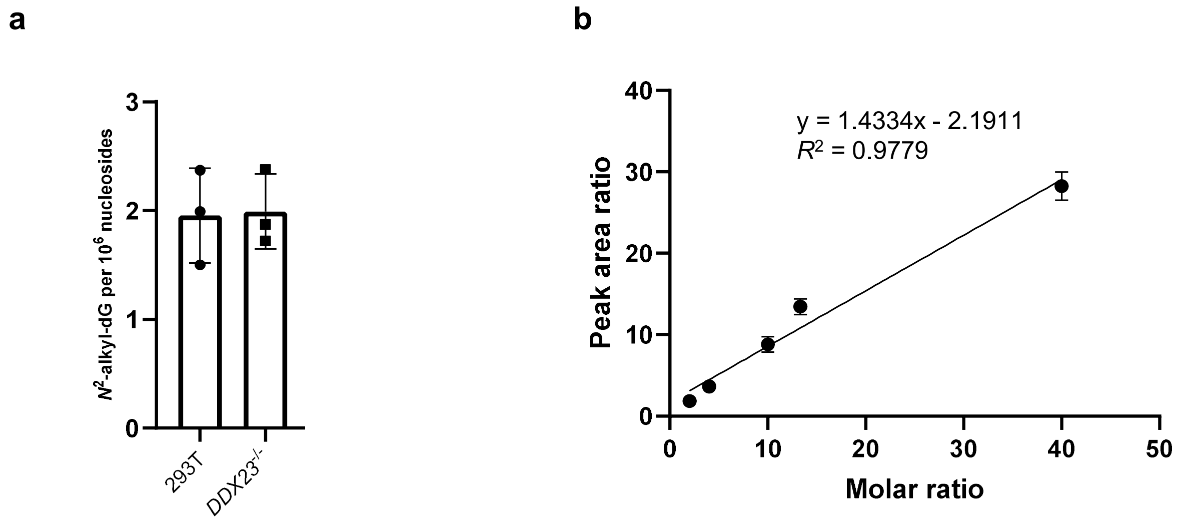

Figure S5. N^2-heptynyl-dG incorporation assay. (A) Frequencies of N^2-heptynyl-dG in cellular DNA isolated from HEK293T and the isogenic *DDX23*$^{-/-}$ cells after treatment with 10 µM N^2-heptynyl-dG for 3 h. (B) A calibration curve for the quantification of N^2-heptynyl-dG. The calibration curve was constructed by plotting the peak area ratios found in the selected-ion chromatograms for the N^2-heptynyl-dG over [D_9]-N^2-*n*Bu-dG vs. the molar ratios of the N^2-heptynyl-dG over [D_9]-N^2-*n*Bu-dG.

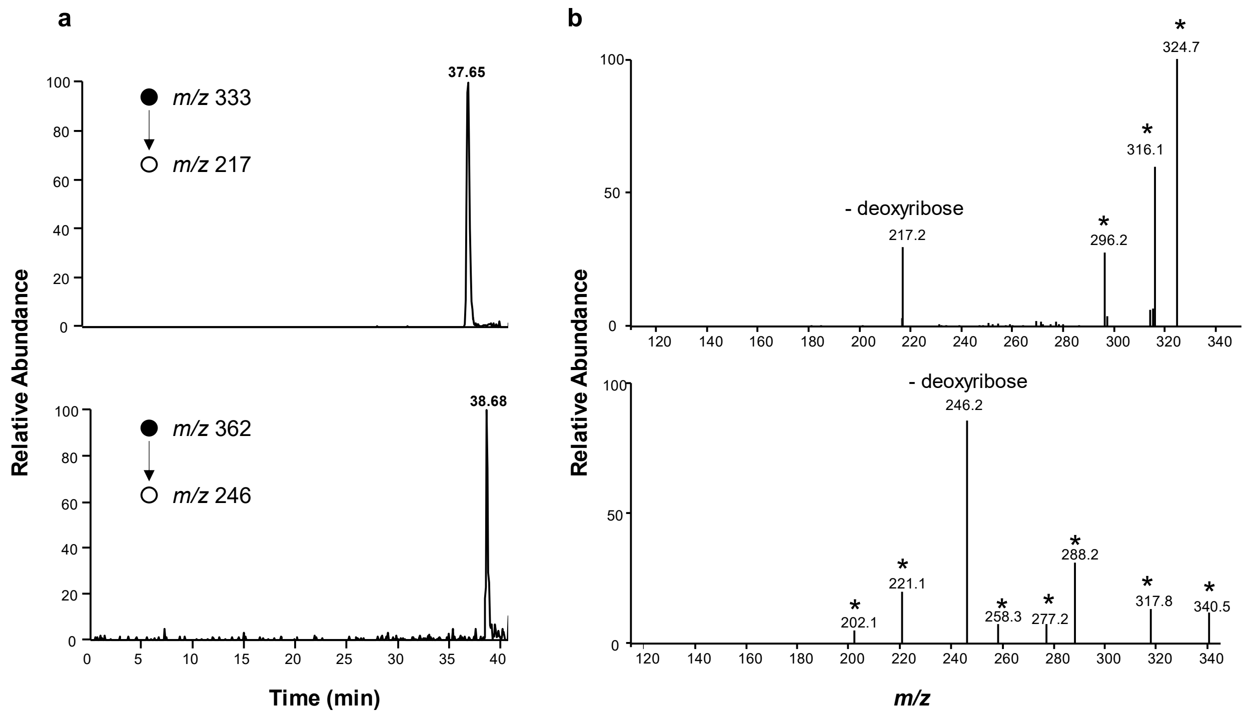

Figure S6. Representative selected-ion chromatograms (SICs) (a) and MS/MS (b) for monitoring the *m/z* 333 → 217 (top panel) and *m/z* 362 → 246 (bottom panel) transitions for the $[M + H]^+$ ions of $[D_9]$-N^2-*n*Bu-dG and N^2-heptynyl-dG, respectively. Ions labeled with "*" are due to the fragmentation of other co-eluting species.

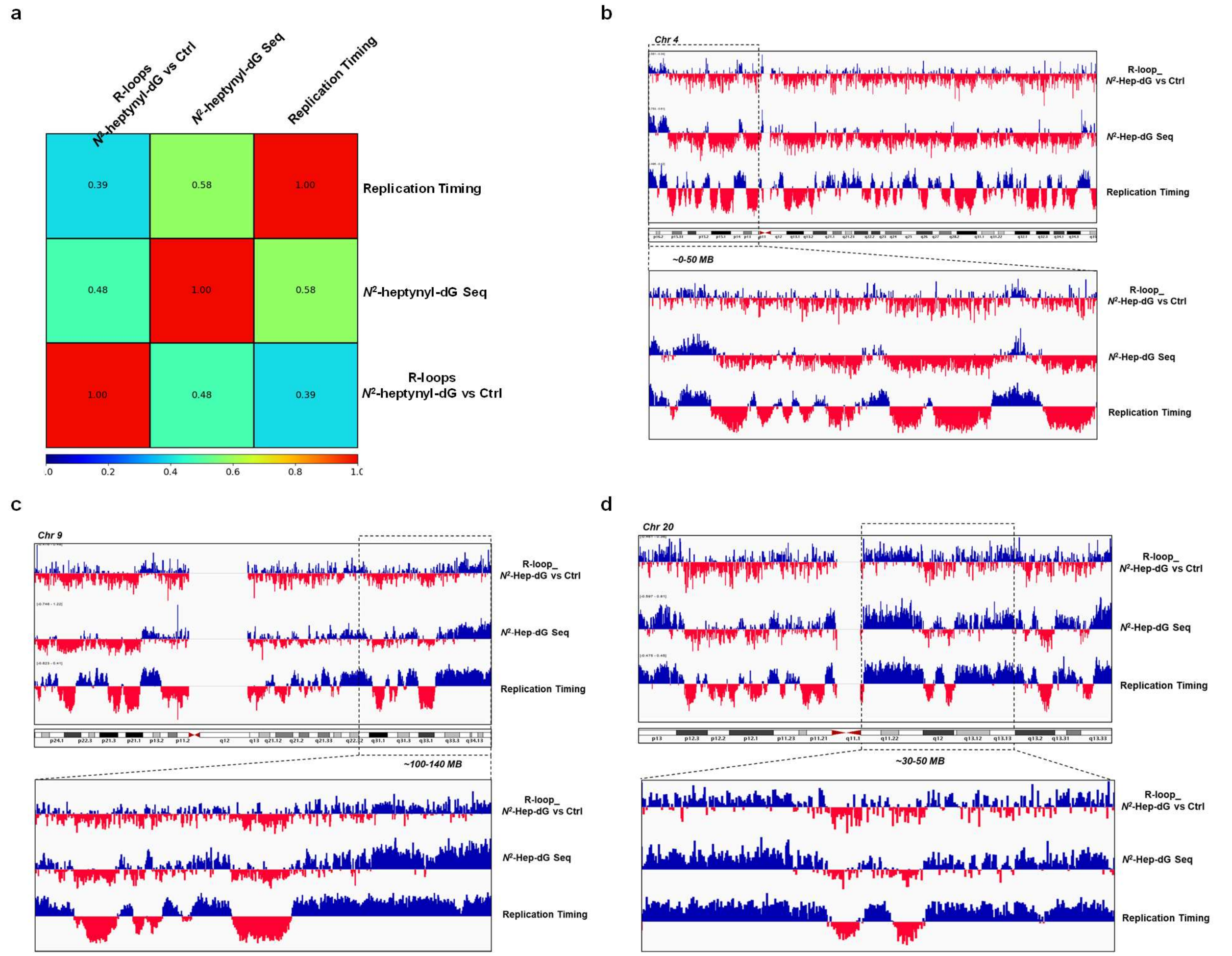

Figure S7. N^2-heptynyl-dG incorporation and N^2-heptynyl-dG-elicited R-loops are co-enriched in genomic regions replicated early in the S phase of the cell cycle. (a) Spearman's correlation coefficients of elevated R-loops, replication timing (PRJNA419407), and N^2-heptynyl-dG-Sequencing (GSE261442) results. (b-d) Representative IGV plots illustrating the comparisons of replication timing, N^2-heptynyl-dG-elicited R-loops, and N^2-heptynyl-dG sequencing results for chromosomes 4, 9, and 20. Two normalized BigWig files were compared using deepTools bigwigCompare, and displayed here are the $\log_2$(ratio) results. The bin size for generating the IGV plots in (b-d) is 50 kb.

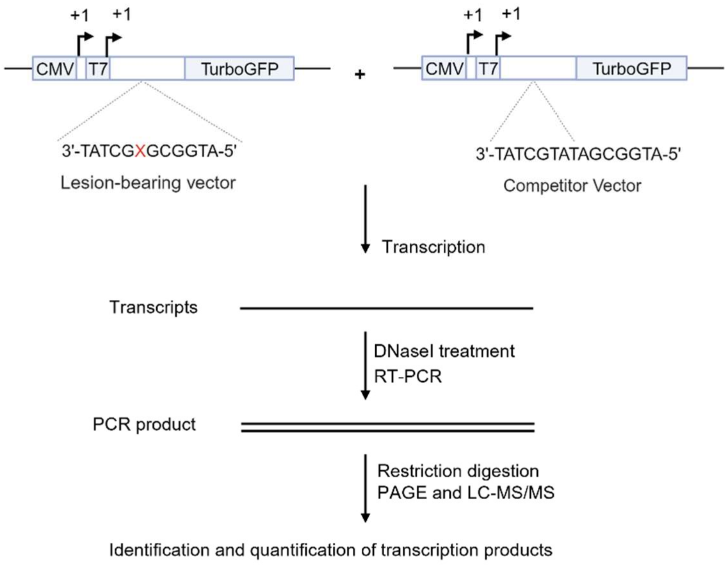

Figure S8. A schematic diagram illustrating the CTAB assay system. 'X' indicates a lesion base or the corresponding unmodified base, which is located on the transcribed strand of the TurboGFP gene downstream of the CMV and T7 promoters. The arrowheads indicate the +1 transcription start sites of the CMV and T7 promoters.

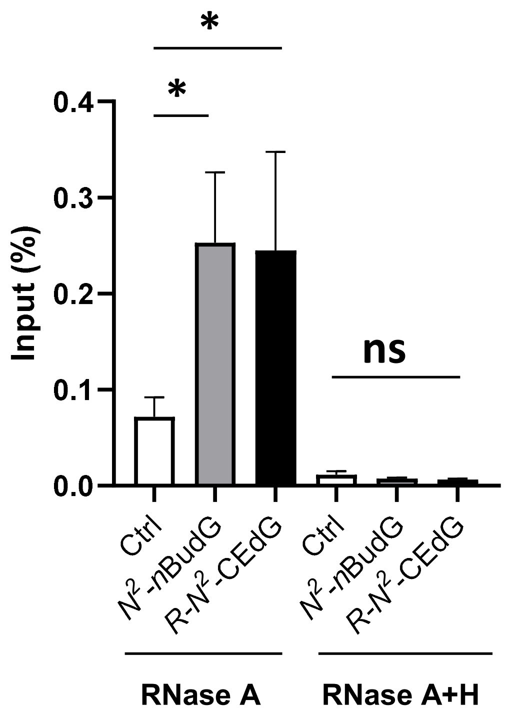

Figure S9. DRIP-qPCR for quantifying the levels of co-transcriptional R-loops formed in transcription from the control and lesion-containing plasmids. Note that RNase H treatment nearly completely abolishes the lesion-induced R-loops. The data represent the mean ± S.D. of results from three replicates. *, $p < 0.05$; ns, $p > 0.05$ (two-tailed Student's t-test).

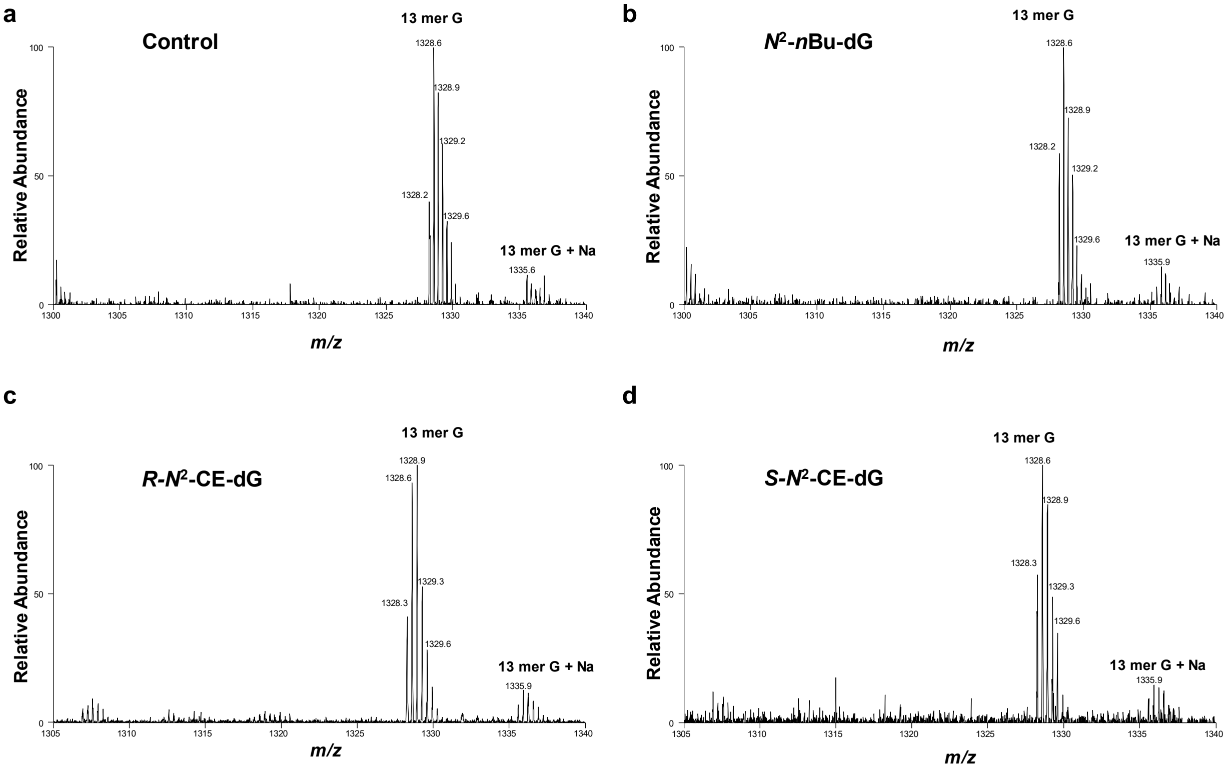

Figure S10. Higher resolution 'ultra zoom-scan' ESI-MS for monitoring the $[M\text{-}3H]^{3-}$ ions of the restriction fragments of interest from transcription of non-lesion control (a) and N^2-alkyl-dG-containing templates (b-d) in *DDX23*$^{-/-}$ cells.

a

DNA	3'-TATCGXGCGGTAC-5'<br>5'-ATAGCCCGCCATG-3'	X = N^2-alkyl-dG
RNA transcript	5'-AUAGCCCGCCAUG-3'	

b

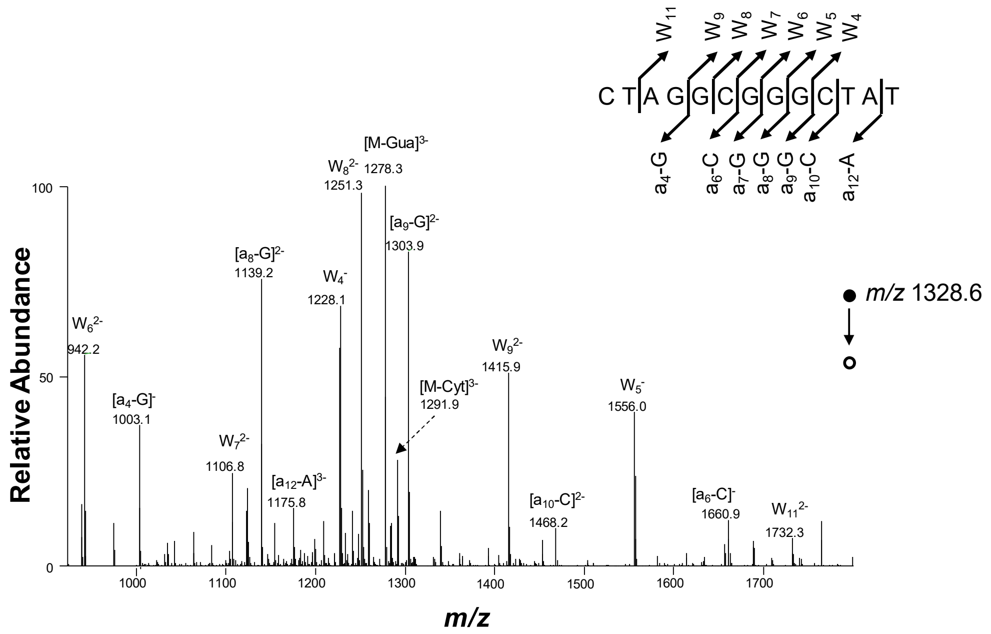

Figure S11. LC-MS/MS for monitoring the restriction digestion products of interest corresponding to wild-type or mutated transcripts arising from N^2-alkyl-dG containing substrates in HEK293T cells. (a) The sequence information of transcript is indicated below the double-stranded DNA construct. (b) The product-ion spectrum of the ESI-produced $[M-3H]^{3-}$ ion (*m/z* 1328.6) of the 13-mer fragments of the wild-type sequence d(CATGGCGGGCTAT).

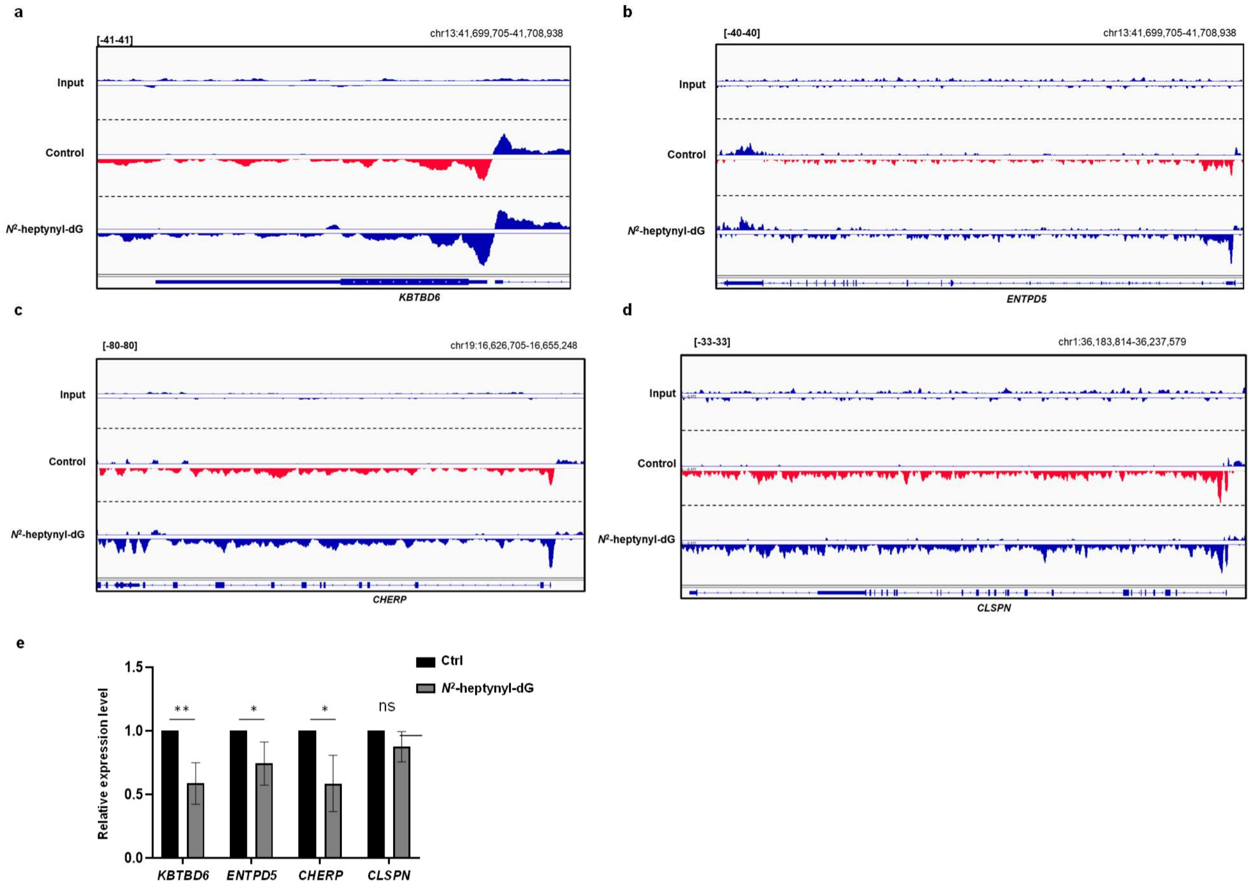

Figure S12. Representative IGV plots showing signal tracks for the spKAS-seq results of *KBTBD6, ENTPD5*, and *CHERP* genes, whose promoters exhibit increased R-loop levels, and *CLSPN* gene, whose promoter displays a similar R-loop level, in N^2-hetynyl-dG-treated HEK293T cells relative to untreated control cells (a-d). (e) RT-qPCR results showing the fold changes in expression levels of *KBTBD6, ENTPD5, CHERP, and CLSPN* genes in N^2-hetynyl-dG-treated and control HEK293T cells. All RT-qPCR results for the indicated genes in N^2-hetynyl-dG-treated cells were normalized to that of *GAPDH*, a house-keeping gene, and the results were further normalized to their respective values observed in untreated control cells. The data represent mean ± S.D. (n = 4). *, $p < 0.05$; **, $p < 0.01$; ns > 0.05 (two-tailed Student's *t*-test).

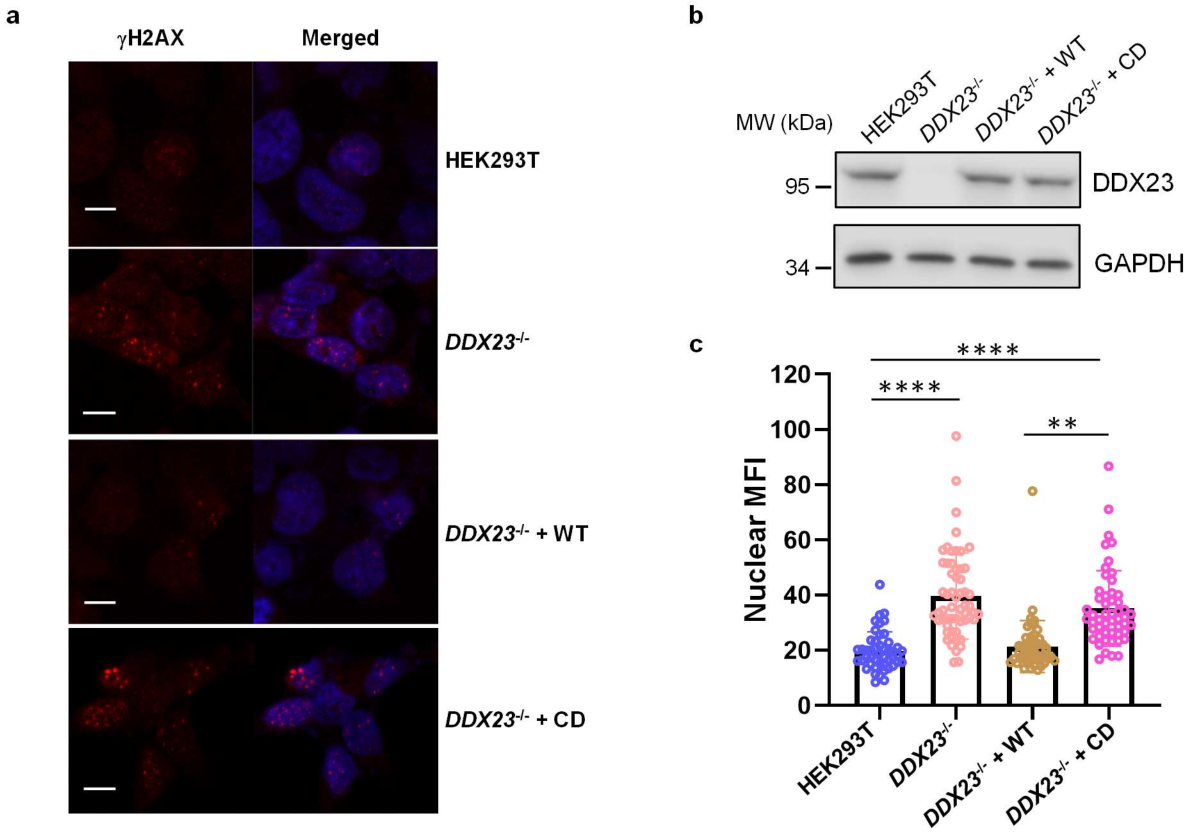

Figure S13. (a) Fluorescence microscopy analysis of γ-H2AX in HEK293T and the isogenic *DDX23*$^{-/-}$ cells with or without overexpressing wild-type (WT) and catalytically dead (CD) DDX23. (b) Western blot for confirming the expression level of DDX23 in HEK293T cells and isogenic *DDX23*$^{-/-}$ cells. (c) Quantification of nuclear γ-H2AX mean fluorescence intensities (MFI) for the conditions shown in (a). The data represent combined results from three biological replicates. **, $p < 0.01$; ***, $p < 0.001$; ****, $p < 0.0001$ (two-tailed Student's *t*-test). Scale bar: 10 μm.
